# Multifrequency magnetic particle imaging enabled by a combined passive and active drive field feed‐through compensation approach

**DOI:** 10.1002/mp.13650

**Published:** 2019-07-16

**Authors:** Dennis Pantke, Nils Holle, Akshay Mogarkar, Marcel Straub, Volkmar Schulz

**Affiliations:** ^1^ Department of Physics of Molecular Imaging Institute for Experimental Molecular Imaging RWTH Aachen University Faculty of Medicine Aachen Germany

**Keywords:** feed‐through suppression, functional parameter estimation, magnetic particle imaging, multifrequency excitation

## Abstract

**Purpose:**

Magnetic particle imaging (MPI) allows fast imaging of the spatial distribution of superparamagnetic iron‐oxide based nanoparticles (SPIONs). Recent research suggests that MPI furthermore promises *in‐vivo* access to environmental parameters of SPIONs as temperature or viscosity. Various medical applications as nanomedicine, stem cell‐based therapies or magnetic hyperthermia could benefit from *in‐vivo* multiparameter estimation by MPI. One possible approach to get access to functional parameters is particle excitation at multiple frequencies. To enable the investigation of the mentioned approach, a novel MPI device capable of multifrequency excitation is needed.

**Methods:**

MPI usually employs analog band‐stop filters to cancel the drive field feed‐through, which is magnitudes higher than the particle signal. To enable drive field frequency flexibility over a wide bandwidth, we propose a combined passive and active drive field feed‐through compensation approach. This cancellation technique further allows the direct detection of the SPIONs' signal at the fundamental excitation frequency.

**Results:**

A combined feed‐through suppression of up to −125 dB is reported, which allows to adjust the drive field frequency from 500 Hz to 20 kHz. Initial spectroscopic measurements and images are shown that demonstrate the concept of multifrequency excitation and prove the imaging capability of the presented scanner. A mean signal‐to‐noise ratio (SNR) enhancement by the factor of 1.7 was shown when the first harmonic is used for measurement‐based image reconstruction compared to when it is omitted.

**Conclusions:**

In this paper, the first one‐dimensional multifrequency magnetic particle imaging (mf‐MPI) that features adjustable excitation frequencies from 500 Hz to 20 kHz is presented. The device will be used to study the principle of multiparameter estimation by employing multifrequency excitation.

## 1 Introduction

Magnetic particle imaging (MPI) is a noninvasive imaging modality that determines the spatial distribution of superparamagnetic iron‐oxide based nanoparticles (SPIONs) *in‐vivo*.^1^, ^2^ It exploits the characteristic nonlinear magnetization function of SPIONs, which leads to a distorted magnetization response to sinusoidal magnetic excitation. Hence, the particle response shows higher harmonics in the frequency domain, rendering the signal distinguishable from the excitation (or *drive*) field feed‐through. MPI provides fast imaging with high spatial resolution and sensitivity, while no ionizing radiation is used. Currently, much effort is made to improve the performance of MPI in regard to these key parameters.^3^, ^4^, ^5^, ^6^ They mainly depend on the hardware of the MPI system, imaging parameters, reconstruction techniques, as well as on the tracer properties.^2^, ^7^ Current commercial preclinical scanners feature a temporal resolution of several tens of milliseconds, a spatial resolution of about 1 mm, and a detection limit of approximately 5–20 ng(Fe).^3^, ^4^

One very promising feature of MPI regarding current biological and medical research and potentially future clinical applications is extracting functional parameters from the SPIONs' local environment. The information on the parameters of interest is encoded in the particle relaxation times *τ*, which can be reconstructed from the magnetic response of the SPIONs to the magnetic excitation field. Several methods to measure temperature or viscosity, distinguish Néel or Brownian contribution or assess cell vitality have been developed.^8^, ^9^, ^10^, ^11^, ^12^, ^13^, ^14^

Various medical applications could benefit from noninvasive *in‐vivo* access to functional parameters as MPI is promising. During applications as magnetic hyperthermia,^15^, ^16^, ^17^ thermally activated drug delivery,^18^ or thermally controlled gene therapy,^19^ *in‐vivo* temperature monitoring is required for dosage control. Information on environmental viscosity and/or the particles' mobility can be employed to distinguish between intra‐ and extracellular sites or assess cell vitality, which may be used for medical applications as nanotherapy or cell tracking during stem cell‐based therapies.^13^, ^14^, ^20^

One promising approach to get access to functional parameters is particle excitation at multiple frequencies.^10^, ^13^, ^21^, ^22^ By multifrequency excitation, the frequency dependence of the relaxation times can be obtained and employed for parameter estimation. The drive field frequency dependence is mainly caused by the different relaxation mechanisms, which are Néel (internal magnetization change) and Brownian relaxation (rotation of bulk material). Recently, Viereck et al. developed a one‐dimensional (1D) scanner that uses two frequencies (10 and 25 kHz) for excitation that are fixed by hardware design to estimate particle mobility.^13^ However, to enhance the access to the mentioned parameters, a signal chain that allows for a wide range of adjustable frequencies is required. Only magnetic particle spectrometers (MPS) that feature this kind of frequency flexibility have been developed.^21^, ^22^, ^23^ To the knowledge of the authors, an MPI scanner that offers a wide range of excitation frequencies and imaging capability has not been published before.

Conventional MPI systems usually employ resonantly powered excitation fields for reactive power handling and filtering of power amplifier (PA) distortions. Additionally, the receive chain usually features a narrow band‐stop filter to cancel the direct drive field feed‐through that is orders of magnitudes higher than the particle signal. The feed‐through would render SPION detection impossible, due to the limited dynamic range of the analog‐to‐digital converter (ADC) as well as possible damage/saturation to the receive electronics.^24^

To enable multifrequency excitation, the mentioned frequency selective approaches have to be avoided. Therefore, we propose a combined passive and active drive field feed‐through compensation approach. Passive precompensation is realized by a custom designed gradiometer‐transmit coil. Then, an active compensation signal is applied via an injection transformer to reduce the residual feed‐through remaining from the passive compensation stage.

Passive cancellation approaches employing gradiometer coils as well as active cancellation techniques have already been implemented in separate systems.^3^, ^22^, ^24^, ^25^ However, the combination of both mentioned feed‐through cancellation approaches in one MPI device to enable multifrequency excitation and imaging is a novel strategy.

The proposed compensation technique allows for broadband excitation and further provides direct access to the fundamental frequency of the particle signal. Thus, the signal‐to‐noise ratio (SNR) can be potentially increased and linearity and shift invariance (LSI) system properties can be maintained without performing fundamental harmonic recovery methods.^26^, ^27^ Furthermore, the combined compensation method allows to simultaneously or sequentially apply multiple frequencies and use the acquired multifrequency data for image reconstruction. The dynamic range of the ADC can be adjusted to the extent of the first harmonic, leading to a minimization of quantization noise.

In this paper, we present the first experimental 1D multifrequency magnetic particle imaging (mf‐MPI) scanner that provides frequency flexibility from 500 Hz to 20 kHz.

## 2 Materials and Methods

### 2.1 System overview

**Figure 1:**
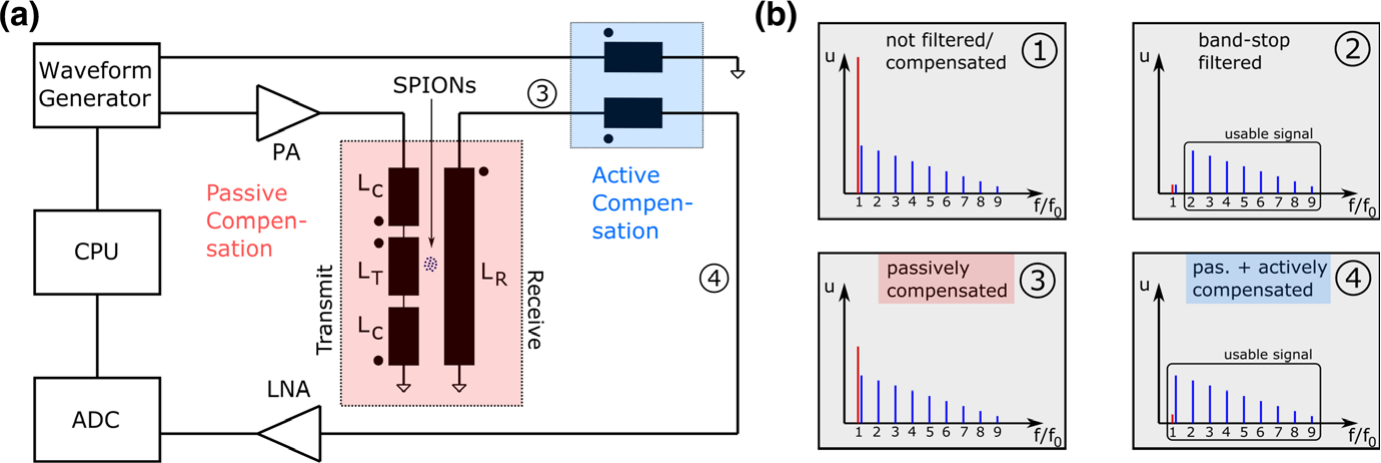
(a) Signal chain of the experimental one‐dimensional multifrequency magnetic particle imaging (mf‐MPI): The excitation signal as well as the compensation signal are computed in software and converted to analog signals by a *Keysight* E33512B waveform generator. The drive field signal is amplified by an *AE Techron* 7796 power amplifier (PA) and then fed into the gradiometric transmit (TX) coil. The sensed superparamagnetic iron‐oxide based nanoparticle (SPION) signal together with the drive field feed‐through pass a low‐loss injection transformer, where the active compensation signal is fed into the receive chain. Afterward, the signal is amplified by a *Stanford research systems* low‐noise‐amplifier (LNA) SR560 and then digitized by an *Adlink* PCIe‐9852 analog‐to‐digital converter (ADC) that is connected to the computation unit (CPU). b) Drive field feed‐through (red) and particle signal (blue) in frequency space of different scanner setups and at different positions of signal chain: 1) Drive field feed‐through is magnitudes higher than the particle signal if it is not filtered or compensated. 2) Received signal when band‐stop filter is used. 3) Signal after passive compensation stage of the mf‐MPI. 4) Signal after passive and active compensation. Direct access to fundamental harmonic of the particle signal is provided. An elaborate description of the active compensation process is given in Section 2.C. [Color figure can be viewed at http://www.wileyonlinelibrary.com]

The signal chain of the proposed 1D mf‐MPI scanner is shown in Fig. 1. The drive field and the active compensation signal is generated by an arbitrary waveform generator *Keysight* E33512B that is remotely controlled by the computation unit (CPU). The drive field signal is amplified by a high‐performance PA *AE Techron* 7796 and then passes the transmit/receive (TX/RX) stage, where the SPION detection and passive precompensation take place. The signal after the passive compensation stage in frequency space can be seen in Fig. 1(b), graph 3. The drive field feed‐through is attenuated, but still exceeds the first harmonic of the particle response. The signal then enters a low‐loss injection transformer, where the active compensation signal is fed into the receive chain. The signal after the active compensation stage is shown in Fig. 1(b), graph 4. The feed‐through is now suppressed below the level of the fundamental harmonic. In difference to scanner setups that use band‐stop filters in the receive chain [Fig. 1(b), graph 2], the first harmonic can be detected directly and used for image reconstruction. After amplification by a *Stanford research systems* preamplifier/low‐noise‐amplifier (LNA) model SR560 the signal is digitized by an *Adlink* PCIe‐9852 ADC that is connected to the CPU. Thus, the signal chain provides a feedback loop, which is required for active compensation.

**Figure 2:**
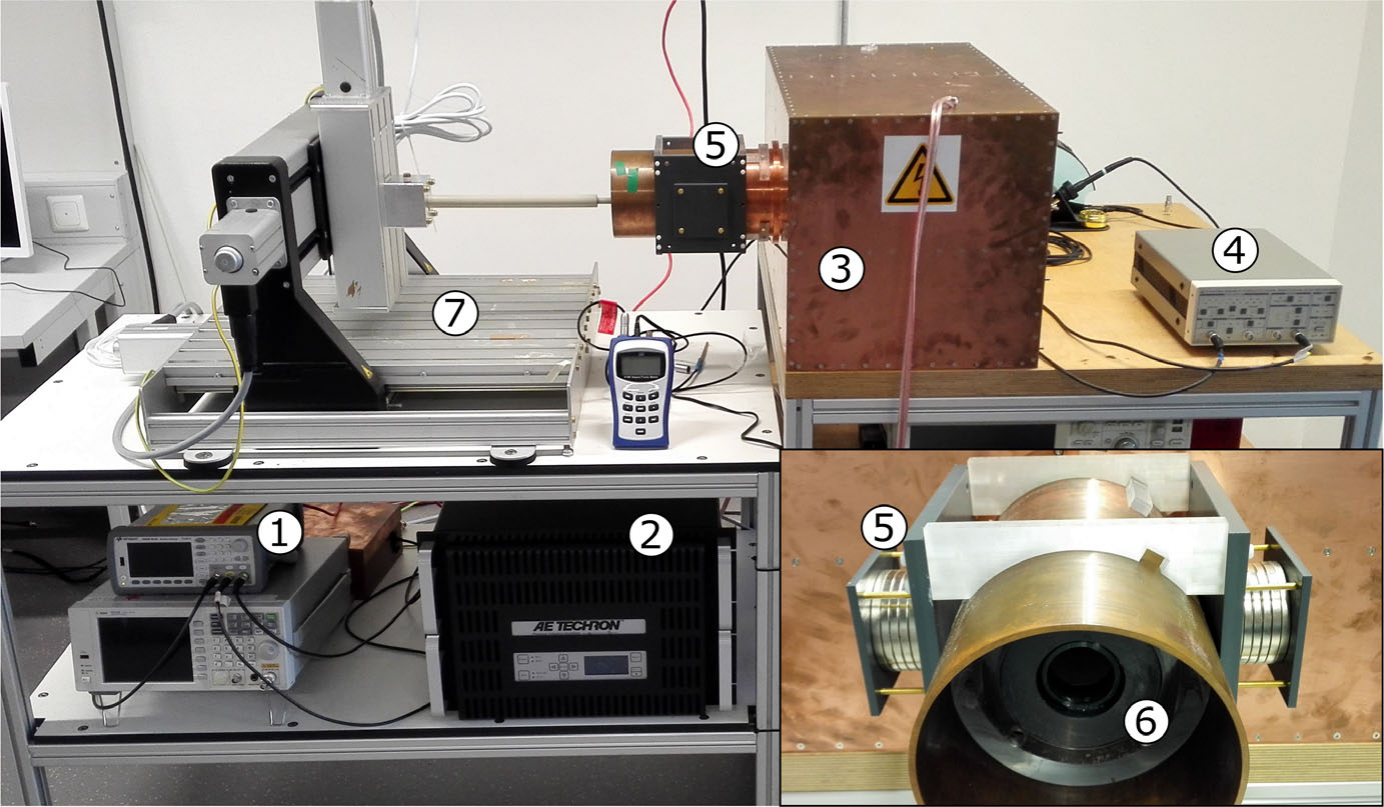
Experimental setup for multifrequency magnetic particle imaging (mf‐MPI): 1) arbitrary waveform generator, 2) power amplifier (PA), 3) shielding cabin, 4) low‐noise‐amplifier (LNA), 5) selection field generating magnet, 6) transmit/receive (TX/RX) coils, 7) robot. [Color figure can be viewed at http://www.wileyonlinelibrary.com]

In order to provide sufficient shielding also at lower frequencies, a shielding cabin of 2 mm thick copper sheets was designed and manufactured (Fig. 2). It contains the TX and RX coils and compensation electronics including the injection transformer. For the acquisition of system matrices as well as accurate positioning of samples, a controllable robot (*isel‐automation*) is used.

The scanner features a linear *x* gradient of 0.7 T/m generated by permanent neodymium magnets, with a 30 mm field of view (FOV) achievable. The magnets are fixed in a mounting partially three‐dimensional (3D)‐printed and partially made of polyvinyl chloride (PVC) placed around the cylindrical copper shield of the TX/RX coil system (Fig. 2).

### 2.2 Passive compensation

**Figure 3:**
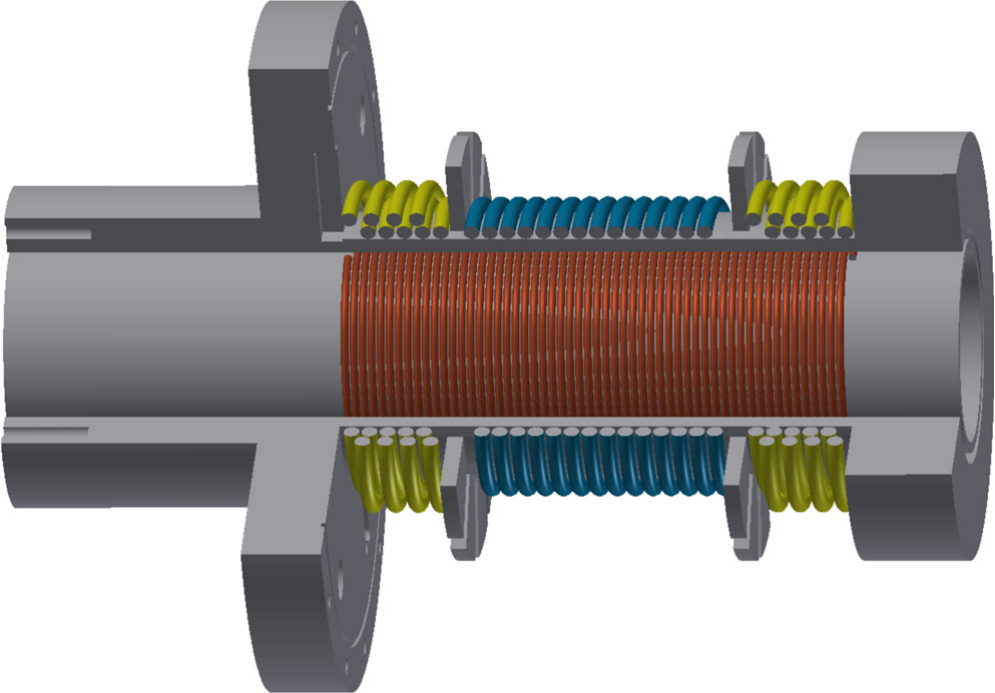
CAD sketch of transmit (TX) and receive (RX) coil design for passive precompensation. The gradiometric TX coil is wound on top of the RX coil $L_{R}$ (red) and subdivided in three sections. The peripheral segments of the TX coil $L_{C}$ (yellow) are wound in two layers and the opposite direction to the central segment $L_{T}$ (blue). [Color figure can be viewed at http://www.wileyonlinelibrary.com]

Passive precompensation of the drive field feed‐through was realized by inductive decoupling of separate transmit (TX) and receive (RX) solenoid coils in a 1D gradiometer coil design (Fig. 3). The inner bore diameter of the solenoid RX coil $L_{R}$ is 33 mm and its length is 70 mm, enabling imaging applications of small animals or phantoms. It is placed inside the gradiometric TX coil and can be moved along the longitudinal (*x*‐)axis to achieve fine tuning. To reduce resistance and avoid skin effect, *Pack* Litz wire of 1.5 and 3 mm diameter was used for the RX coil and TX coil, respectively. The gradiometric TX coil is realized as a three‐section arrangement.^28^, ^29^ The peripheral segments $L_{C}$ are wound in the opposite direction to the central segment $L_{T}$. Hence, they induce a voltage in the receive coil, which cancels out the voltage induced by $L_{T}$.

The coupling between the TX and RX coil was minimized to maximize the feed‐through cancellation. Therefore, the mutual inductance was minimized in the thin‐wire approximation:

(1) $$M\propto \underset{=:M_{0}}{\underbrace{\oint_{\partial A_{T}}\oint_{\partial A_{R}}\frac{dr_{T}\cdot dr_{R}}{||dr_{T}-dr_{R}\left|\right|}}}-\underset{=:M_{1}}{\underbrace{\oint_{\partial A_{C}}\oint_{\partial A_{R}}\frac{dr_{C}\cdot dr_{R}}{||dr_{C}-dr_{R}\left|\right|}}}$$

where $M_{0}$ is the mutual inductance between the transmit section $L_{T}$ and $L_{R}$, $M_{1}$ is the mutual inductance between the cancellation section $L_{C}$ and $L_{R}$ and $A_{C}$, $A_{T}$, as well as $A_{R}$ are the cross sections of the respective coils. Fine tuning was achieved by connecting the coils to a network analyzer *Keysight* E5061B and adjusting the position of the RX coil until the power transfer between TX and RX was minimized. A power transfer measurement with disconnected cancellation part $L_{C}$ served as a reference and was subtracted from the measured power transfer.

### 2.3 Active compensation

A custom‐made low‐loss injection transformer placed outside the bore but inside the shielding cabin is used to actively apply a compensation signal that removes the drive field feed‐through remaining from the passive compensation stage. The transformer is made of 1.5 mm thick Litz wire. The inductance relation from primary to secondary coil is 24.9 to 23.4 *μ*H. The measured coupling factor of the transformer is 0.88. As the current prototype does not include active cooling of the coils, pulsed mode operation has been chosen to avoid overheating. However, the coils and the shielding cabin are designed to support active cooling, which will be implemented in the future to enable continuous mode operation.

For cancellation, the active compensation signal needs to resemble the inverse of the feed‐through after passive compensation, which shows nonlinear amplitude and phase drifts mainly due to heating of electrical components. Therefore, an active feedback of the feed‐through after both compensation stages is needed. For that purpose, an active compensation control (ACC) was implemented that allows to minimize the residual feed‐through, which is measured at the input of the ADC. One iteration of the ACC is illustrated in Fig. 5(a). In the current prototype, the active cancellation signal is updated after every particle scan. The scan duration can be set by software. Prior to each scan, the robot moves the sample out of the bore and a compensation measurement (calibration) is performed. This is an empty measurement, which is used to determine the active compensation signal that cancels the actual drive field feed‐through including the drift. Since the feed‐through after only passive compensation would still saturate the receive electronics, disturbing the ACC algorithm and leading to possible damage of the LNA, an initial compensation signal is necessary. The initial compensation signal was determined once in a calibration, for which the drive field frequency was varied. Since only the residual feed‐through after both compensation stages $V_{m}^{n}\left(\omega ,\varphi_{m}^{n}\right)$ can be measured, the actual drive field feed‐through $V_{D}^{n}\left(\omega ,\varphi_{D}^{n}\right)$ is calculated from the known applied initial compensation signal $V_{C}^{n}\left(\omega ,\varphi_{C}^{n}\right)$:

(2) $$V_{D}^{n}\left(\omega ,\varphi_{D}^{n}\right)=V_{m}^{n}\left(\omega ,\varphi_{m}^{n}\right)-V_{C}^{n}\left(\omega ,\varphi_{C}^{n}\right).$$

Finally, the calculated feed‐through $V_{D}^{n}\left(\omega ,\varphi_{D}^{n}\right)$ after only passive compensation is used to derive the compensation signal for the next period $V_{C}^{n+1}\left(\omega ,\varphi_{C}^{n+1}\right)$ by applying a 180${}^{\circ }$ phase shift:

(3) $$V_{C}^{n+1}\left(\omega ,\varphi_{C}^{n+1}\right)=V_{D}^{n}\left(\omega ,\varphi_{D}^{n}+\pi \right).$$

The algorithm is further optimized by adding the phase drift between two pulses $\Delta \varphi \;=\;\varphi_{D}^{n+1}\;-\;\varphi_{D}^{n}$ interpolated from the previous iterations:

(4) $$V_{C}^{n+1}\left(\omega ,\varphi_{C}^{n+1}\right)=V_{D}^{n}\left(\omega ,\varphi_{D}^{n}+\pi +\Delta \varphi \right).$$

### 2.4 Spectroscopic measurements

To prove the concept and functionality of multifrequency excitation with the presented mf‐MPI, initial spectroscopic measurements (no selection field) were performed. Frequency spectra of a 100 *μ*l Perimag^®^ (*micromod Partikeltechnologie GmbH*) sample with an iron concentration of 8.5 mg/ml were acquired. Perimag^®^ consists of clustered core particles with a hydrodynamic diameter of 130 nm including a dextran coating. The drive field frequency was varied between 1 and 20 kHz in 1 kHz steps and the drive field strength was decreased linearly from 10 mT at 1 kHz to 5 mT at 20 kHz. The sample was placed at the tip of a rod connected to the robot and positioned in the center of the FOV.

### 2.5 Imaging measurements

To demonstrate the imaging capability of the presented device, phantom images were acquired. Several drive field frequencies were used for acquisition; these were 5, 7, 10, 13, and 15 kHz. Each image was acquired five times for averaging. The phantom consists of two samples longitudinally aligned along the *x*‐axis of the bore. The dimensions of the samples are 1 × 1 × 2 mm (*x*,*y*,*z*), corresponding to 2 *μ*l each. The distance between the sample centers is 3 mm. Perimag^®^ with an iron concentration of 8.5 mg/ml was used. The applied sinusoidal drive field amplitude of the respective excitation frequency was 7 mT. The 3D‐printed phantom is designed to easily be pulled over the tip of the rod that is mounted on the robot. Measurement‐based image reconstructions were performed by acquiring system matrices and employing the Kaczmarz method.^30^ The system matrices vary in the applied drive field frequencies. They were generated using a drive field strength of 7 mT. The sampling grid featured 49 sampling positions with 0.5 mm space between each position leading to a FOV of 24 mm. The dimensions of the Perimag^®^ delta sample were 1 × 1 × 2 mm (*x*,*y*,*z*). The reconstruction parameters were kept constant for all reconstructions of the phantom. 50 iterations were used, the regularization parameter was set to zero and all frequency components besides the harmonics of the excitation signals were omitted.

## 3 Results

### 3.1 Passive compensation

**Figure 4:**
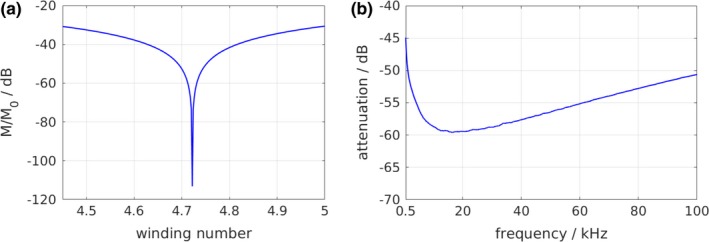
(a) Calculated mutual inductance *M* divided by $M_{0}$ as a function of the winding number *n* of the outermost layer of $L_{C}$. A thin‐wire approximation (1) was used for computation. Due to manufacturing reasons, the winding number was rounded up to 5. (b) Broadband feed‐through attenuation by passive decoupling using a gradiometric coil design: Power transfer TX‐RX measured by a network analyzer (differential mode). The measured power transfer was subtracted by a reference measurement for which the cancellation coils were disconnected. [Color figure can be viewed at http://www.wileyonlinelibrary.com]

Figure 4(a) shows the computed mutual inductance *M* divided by $M_{0}$ as a function of the winding number of the outermost layer of $L_{C}$. The winding number minimizing the coupling that results from the calculation was rounded up due to manufacturing reasons and the TX coils were wound in accordance to that. Fine tuning was performed by adjusting the position of the RX coil relative to the TX coil. The measured power transfer between TX and RX subtracted by the reference measurement is depicted in Fig. 4(b). In the frequency range from 0.5 to 100 kHz, the drive field feed‐through attenuation is between −45 and −59 dB. The maximum suppression can be observed at approximately 16 kHz. The frequency dependence is discussed in Section 4

### 3.2 Active compensation

**Figure 5:**
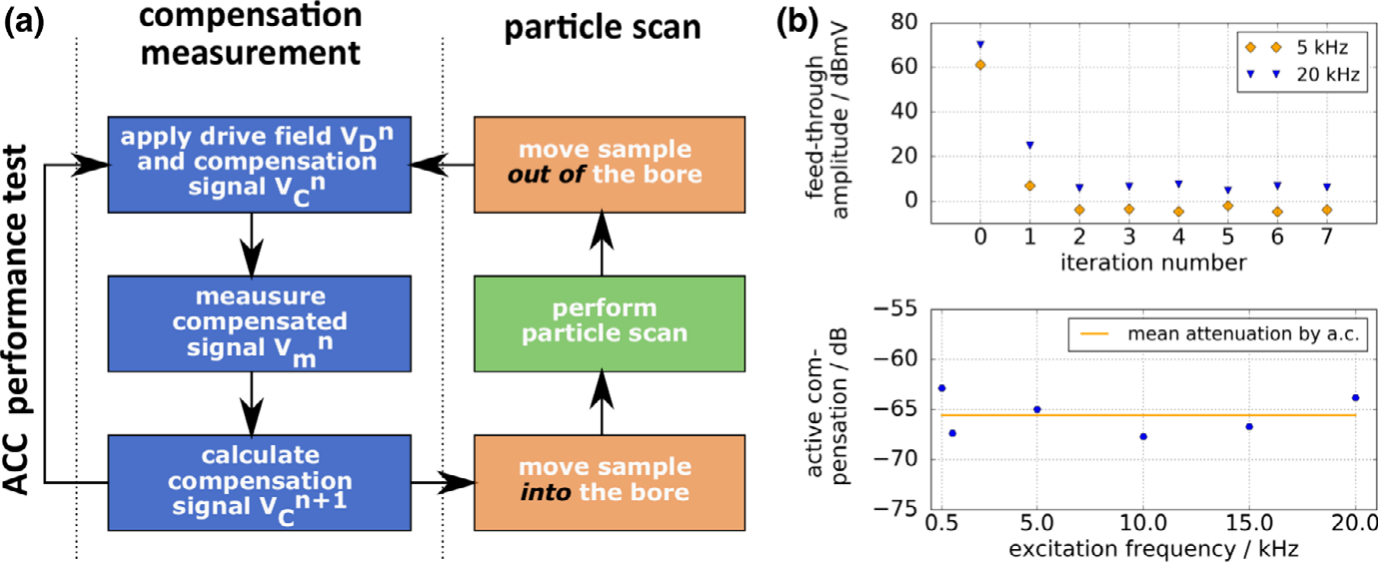
(a) Flowchart of one iteration of the active compensation control (ACC). The compensation signal for the next iteration is calculated from the measured compensated signal after both compensation stages and the known applied compensation signal. Then, the sample is moved into the bore and a particle scan is performed. The compensation signal is updated after each particle scan. During the ACC performance test, no particle scans were performed. (b) Top: Convergence of the ACC exemplarily shown for two excitation frequencies: 5 and 20 kHz. At each drive frequency, eight iterations were performed and the ACC was applied five times for averaging. The residual feed‐through reaches a steady state after maximum two iterations. Bottom: Feed‐through suppression as difference of initial iteration 0 and mean of iterations 2–7. The measured mean attenuation was −66 ± 2 dB. [Color figure can be viewed at http://www.wileyonlinelibrary.com]

The additional suppression achieved by active compensation was determined by running eight iterations of the ACC and measuring the difference of the feed‐through at iteration 0 (here the compensation signal amplitude is set to zero; the LNA gain was reduced to prevent the receive electronics from saturation) and after active compensation (mean of iteration 2–7) was applied. The drive field pulse duration was 20 ms and the amplitude was 5 mT. This was done for several frequencies between 0.5 and 20 kHz. Each ACC run was repeated five times for averaging. Figure 5(b) top shows the convergence of the ACC for the two applied drive frequencies: 5 and 20 kHz. The residual feed‐through reaches a steady state after maximum two iterations. An additional reduction of the feed‐through between −63 (0.5 kHz) to −68 dB (10 kHz) was measured. The mean additional feed‐through suppression by active compensation was −66 ± 2 dB. This is depicted in Fig. 5(b) bottom. In total, attenuation values up to −125 dB were achieved (Table 1).

**Table 1:** Drive field feed‐through attenuation by passive, active, and combined passive and active compensation (total)

Frequency	Passive compensation	Active compensation	Total
0.5 kHz	−45 dB	−66 ± 2 dB	−111 ± 2 dB
1 kHz	−50 dB	−66 ± 2 dB	−116 ± 2 dB
5 kHz	−56 dB	−66 ± 2 dB	−122 ± 2 dB
10 kHz	−59 dB	−66 ± 2 dB	−125 ± 2 dB
15 kHz	−59 dB	−66 ± 2 dB	−125 ± 2 dB
20 kHz	−59 dB	−66 ± 2 dB	−125 ± 2 dB

### 3.3 Spectroscopic measurements

**Figure 6:**
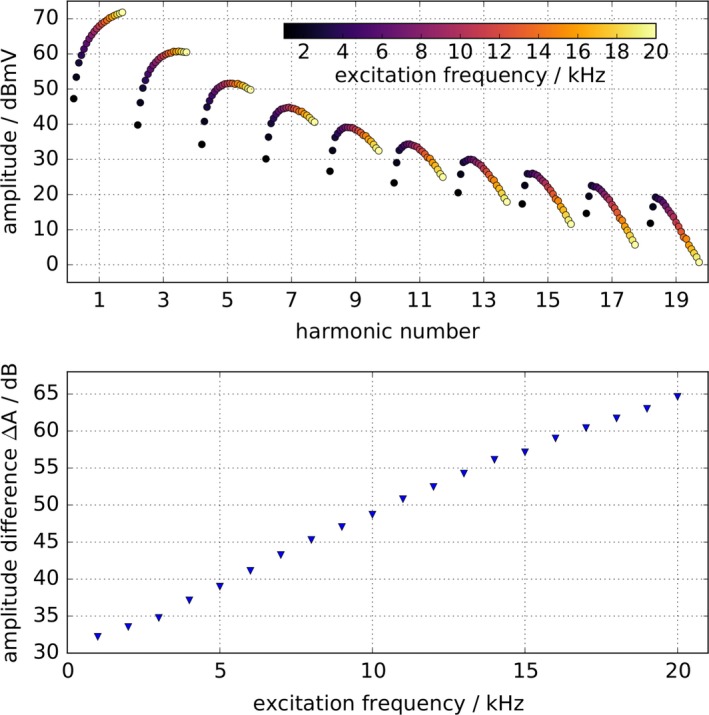
Top: odd harmonics of a 100 *μ*l Perimag^®^ sample generated by applying various drive field frequencies between 1 and 20 kHz. The excitation frequency is color encoded. The amplitudes are corrected by the receive chain characteristics. The drive field amplitude was decreased linearly from 10 mT at 1 kHz to 6 mT at 20 kHz. Bottom: amplitude difference Δ*A* between the first and 19th harmonic dependent on the drive field frequency. The amplitude drop increases linearly on a logarithmic scale. [Color figure can be viewed at http://www.wileyonlinelibrary.com]

Figure 6 top shows the odd harmonics that are generated by various spectroscopic acquisitions of a 100 *μ*l Perimag^®^ sample. All measurements throughout the applied excitation frequencies are depicted in one graph by placing the values belonging to one specific harmonic next to each other. The excitation frequency is color encoded. The noise floor is at approximately −10 dBmV, while no averaging was applied. Although the excitation field is decreasing linearly, a signal increase of the fundamental harmonic can be observed for rising frequencies, resulting primarily from Faraday's law of induction. Furthermore, the harmonics are converging faster with increasing excitation frequency. To show the influence of the excitation frequency on the decay of the higher harmonics, the amplitude difference between 1st and 19th harmonic $\Delta A\;=\;A^{1}\;-\;A^{19}$ is depicted in Fig. 6, bottom. The amplitude drop increases linearly on logarithmic scale from 32 dB at 1 kHz to 65 dB at 20 kHz.

**Figure 7:**
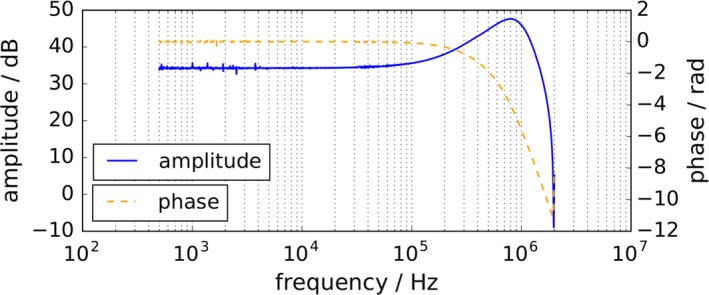
Power transfer function (blue) of the multifrequency magnetic particle imaging (mf‐MPI) receive chain [receive (RX) coil–low‐noise‐amplifier (LNA)] and phase of receive chain (orange). The LNA features an internal low‐pass filter, which was set to 1 MHz. Apart from the filter behavior the receive chain shows a very uniform gain between 500 Hz and approximately 100 kHz. To show that the drive frequency‐dependent increase in signal drop between first and 19th harmonic (Fig. 6) is not caused by the receive chain, the results of the spectroscopic measurements were corrected by these data. [Color figure can be viewed at http://www.wileyonlinelibrary.com]

To exclude a frequency‐dependent influence of the receive chain on the harmonic spectrum, the power transfer of the receive chain was measured and used to correct the results of the spectroscopic measurements by these data. Figure 7 depicts the absolute power transfer (blue) of the mf‐MPI receive chain (RX coil–LNA) and the phase of the receive chain (orange). A small pick‐up coil connected to the output port of a network analyzer was used to determine the receive chain power transfer. The pick‐up coil was driven into the center of the RX coil. The output of the LNA was connected to the input port of the network analyzer. The coupling between the pick‐up coil and RX coil was determined separately and subtracted from the overall power transfer (pick‐up coil–RX coil–LNA). The LNA features an internal low‐pass filter, which was set to 1 MHz. This can be observed in the power transfer plot. Apart from that, the receive chain shows a very uniform gain between 500 Hz and approximately 100 kHz.

### 3.4 Imaging measurements

**Figure 8:**
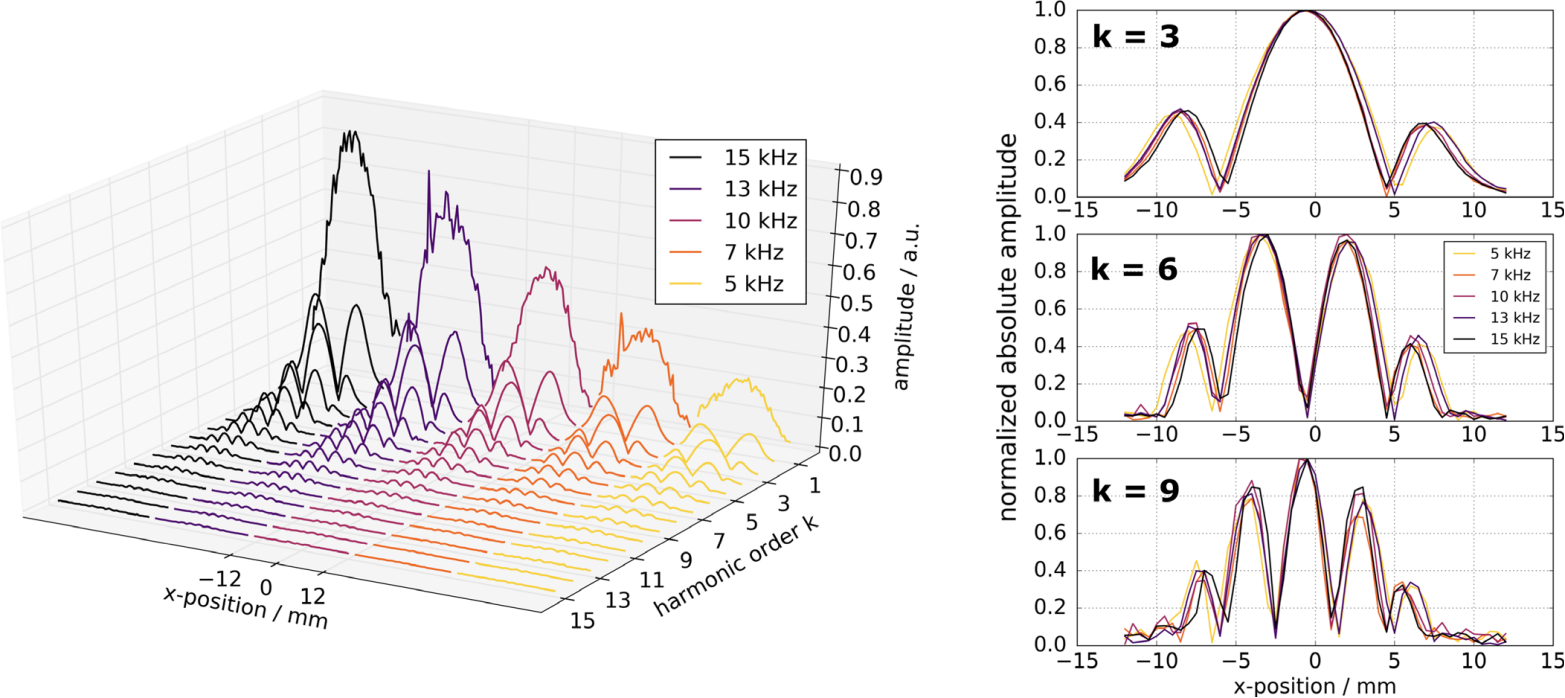
Left: absolute value of measured system matrices at the first 15 harmonic orders. Five system functions differing in the applied excitation frequency are displayed next to each other on the *x*‐axis. The first order *k* = 1 indicates the fundamental frequency with respect to the excitation. The respective higher harmonics are placed in front to each other. The *x*‐axis labels the *x*‐position im mm for a specific excitation frequency and is repeating for each system function. The fundamental frequencies appear more noisy than the higher frequency components. This is addressed in Section 4, discussion. Right: normalized magnitudes of frequency components *k* = 3, 6 and 9. The system matrices corresponding to the respective drive frequencies show differences apart from only the absolute intensities. [Color figure can be viewed at http://www.wileyonlinelibrary.com]

Figure 8 left shows the absolute values of the measured system functions at the first 15 frequency components in a 3D plot. The *y*‐axis labels the frequency components. *k* = 1 indicates the fundamental frequency. The *x*‐axis labels the *x*‐position of the delta sample. The system matrices varying in the applied drive frequencies are plotted next to each other on the *x*‐axis and are color encoded. As already observed in the spectroscopic measurements, the signal drop between first and seventh harmonic is bigger for increasing excitation frequencies. Note, that also the system function's first order is displayed, which cannot be done with single excitation frequency devices. For particles with an ideal Langevin behavior, the 1D system function can be described by the Chebyshev polynomials of the second kind.^5^, ^31^ This pattern can be observed in the plots.

In Fig. 8 right, the normalized magnitudes of the frequency components *k* = 3, 6, and 9 are exemplarily depicted for each drive frequency. Aside from the absolute amplitudes, the system matrices at the respective harmonic orders slightly differ from each other. Depending on the excitation frequency, they are slightly compressed or stretched.

**Figure 9:**
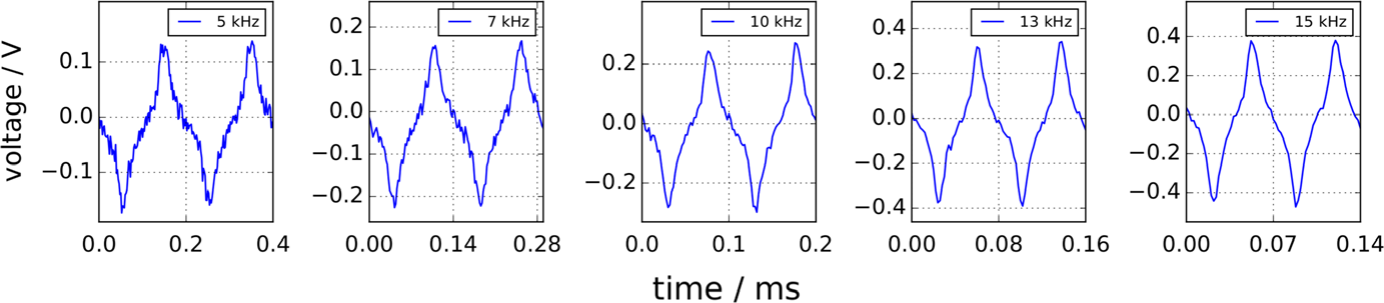
Raw time domain data of phantom image acquisitions at different frequencies as detected by the analog‐to‐digital converter (ADC). Two cycles are shown for each individual acquisition. The fundamental frequency can already be identified in the raw time domain data. [Color figure can be viewed at http://www.wileyonlinelibrary.com]

**Figure 10:**
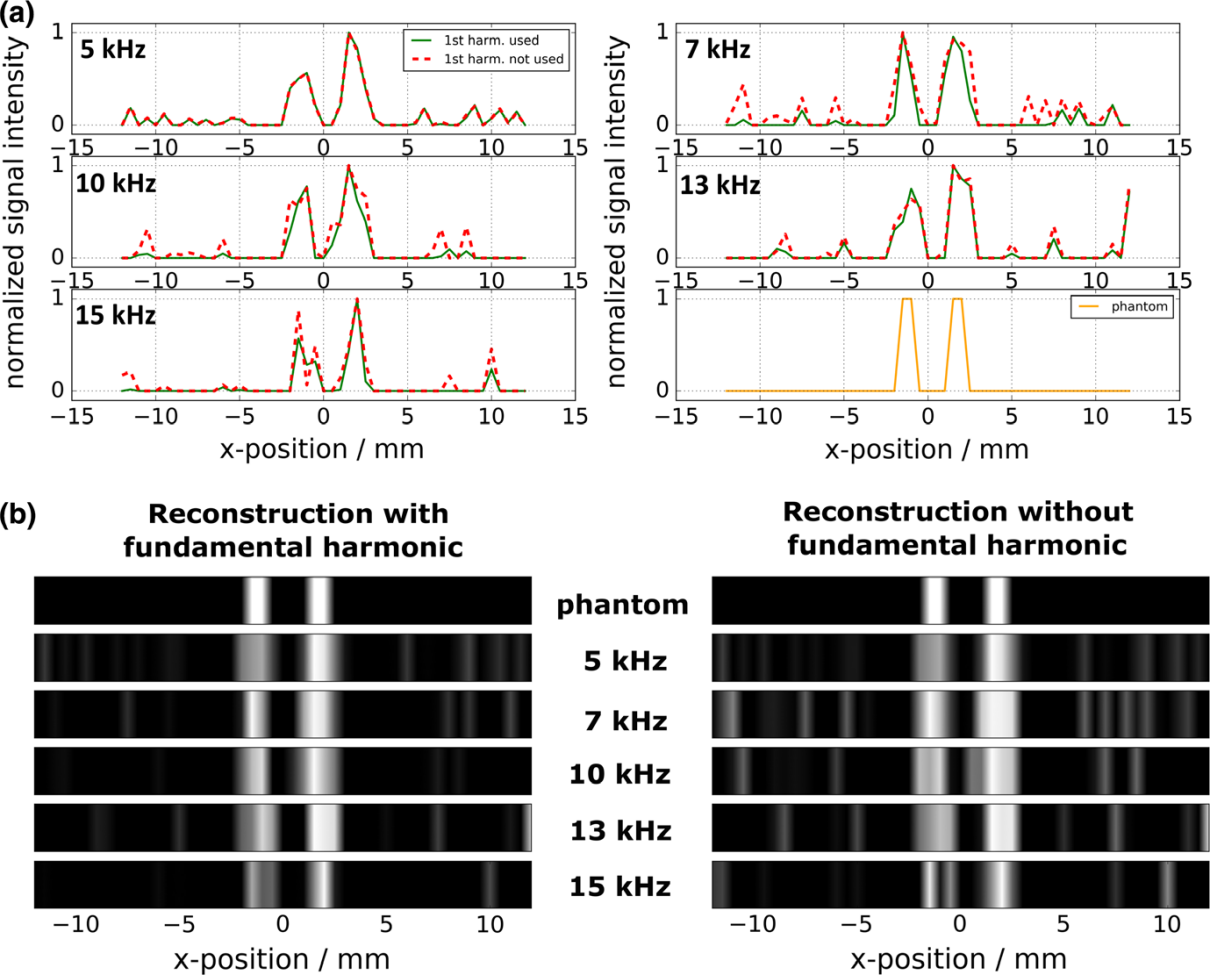
Comparison of phantom image reconstruction with and without using the system function's fundamental harmonic. The applied drive field frequencies were 5, 7, 10, 13, and 15 kHz. The various reconstructions were performed on the same initial raw data. (a) Line profile representations of image reconstructions and actual phantom pattern individually plotted for each applied drive field frequency. (b) Gray‐value images. The signal‐to‐noise ratio (SNR) is decreased when the fundamental harmonic is omitted in the reconstruction process. [Color figure can be viewed at http://www.wileyonlinelibrary.com]

Figure 10 shows a comparison of phantom image reconstructions with and without using the system function's fundamental harmonic. Five different excitation frequencies were applied and the respective acquired system matrices were employed for image reconstruction: 5, 7, 10, 13, and 15 kHz. The line profiles along the *x*‐axis (a) as well as the gray value images (b) are shown. Both are normalized to the respective maximum intensity of each acquisition. The phantom images could be reconstructed successfully with all applied drive frequencies. When the fundamental frequency is omitted in the reconstruction process, an overall SNR decrease from 15.2 to 8.8 can be observed. Hence, the SNR was increased by a factor of 1.7 when the fundamental harmonic was employed. The SNR was quantified by defining regions of signal (line profile amplitude of “true” phantom — seen in Fig. 10(a), bottom right — is one) and regions of noise (amplitude is zero) for each sample peak in each image and dividing the mean values. The SNR values of the individual reconstructions can be seen in Table 2. The raw time domain data are shown in Fig. 9.

**Table 2:** SNR values of multifrequency reconstructions with and without employing the fundamental harmonic and the absolute difference. The SNR was calculated by defining regions of signal and regions of noise according to the phantom and dividing the mean values

Excitation frequency	SNR with first harmonic	SNR without first harmonic	Absolute difference
5 kHz	9.9	9.3	0.6
7 kHz	16.5	6.8	9.7
10 kHz	14.9	8.0	6.9
13 kHz	9.2	7.9	1.3
15 kHz	25.7	12.8	12.9
mean	15.2	8.8	6.4–ratio:1.72

## 4 Discussion

The gradiometer coil dimensions and winding numbers were determined by minimizing the coil's mutual inductance [Fig. 4(a)]. Only little fine tuning by adjusting the position of the RX coil with respect to the TX coil was necessary until the power transfer between TX and RX was minimized and a passive compensation up to −59 dB was measured (Fig. 4). The frequency dependence results from capacitive coupling between the two coils and the frequency‐dependent nonuniform current distribution along the wire in each coil. The suppression values reported by other research groups using gradiometer coils for passive decoupling are comparable.^3^, ^22^

The implemented ACC allows effective cancellation of the fundamental harmonic in the currently used pulsed mode operation. Figure 5 shows that only two iterations are necessary to reach a steady state and suppress the drive field feed‐through by up to −66 dB in average.

A total cancellation of the drive field feed‐through up to −125 dB was achieved (Table 1). Eighth‐order band stop filters are theoretically able to suppress the drive field frequency band by up to 160 dB.^24^, ^32^ Thus, the combined feed‐through suppression that is reported here is close to the damping by analog filters. Finally, it is sufficient to allow MPI applications.

However, there is still some potential to optimize the algorithm in terms of absolute suppression: Since the residual feed‐through is measured at the ADC and only the active compensation signal at the output of the waveform generator is known, it is necessary to determine the transfer function of the active compensation signal chain. In addition, small amplitude and phase drifts are causing an error in the calculation of the active compensation signal. This is why a PID‐controller based algorithm for which no transfer functions are needed will be implemented in the next version of the mf‐MPI. This will probably result in an increased feed‐through suppression by active compensation.

As the presented compensation technique is a broadband cancellation approach, it provides excitation frequency flexibility. This was shown by generated frequency spectra acquired with excitation frequencies ranging from 1 to 20 kHz that are depicted in Fig. 6. Basically, two observations can be made coming along with increasing excitation frequency that can be explained as follows: First, the gain of the respective harmonic's amplitude results from Faraday's law of induction. Second, the signal drop between 1st and 19th harmonic is most likely caused by relaxation effects. The influence of the relaxation is getting more dominant with rising frequency, since the difference between relaxation time and cycle duration increases. A very uniform gain of the receive chain can be observed across the frequency band from 500 Hz to approximately 100 kHz in Fig. 7. Since the data of the spectroscopic measurements are corrected by the receive chain characteristics, one can exclude the RX chain as a reason for the increasing signal drop between first and 19th harmonic observed in Fig. 6. The resonance at approximately 800 kHz, which is caused by the low pass filter is in addition beneficial to enhance the SNR of higher harmonics.

In the current scanner setup, arbitrary frequencies up to approximately 20 kHz can be chosen for excitation. The magnetic field strength and applicable frequency range are currently limited by the performance of the PA. Since a reactive load and no band‐pass filter is used in the transmit chain, one must deal with harmonic PA distortions, which are getting more prominent with increasing frequency and output power corresponding to the drive field strength. For excitation frequencies below 20 kHz, the distortions are in the range of 120 dB less than the excitation signal. Thus, the SPIONs' response is not considerably influenced by the distortions. Currently, the maximum drive field is approximately 10 mT at 10 kHz and 6 mT at 20 kHz. A new PA will be chosen in future to increase the applicable drive field frequency and amplitude.

The generated system matrices (Fig. 8) visualize the direct access to the fundamental frequency. Compared to all other higher harmonics, the noise of first harmonic of the system matrices is increased. The reason for this is the drive field drift between the background measurement and the actual measurement, which is not negligibly small. In addition, the absolute values of the system matrices slightly differ in their patterns. This can be observed in Fig. 8, right. The matrices are slightly stretched or compressed depending on the applied drive field frequency. The factor of stretching/compression seems to be uniform throughout the frequency components with respect to one specific drive field frequency. Hence, the drive frequency modulates the system function, which supports the usability of mf‐MPI for multiparametric MPI. A higher viscosity of the delta sample would probably reveal even greater differences of the system functions since it would impede the Brownian rotation.^13^

The loss of the particle signal at the fundamental frequency due to band‐pass filtering is a considerable issue both when doing measurement‐based image reconstruction by a system function^5^, ^33^ or x‐space reconstruction.^26^, ^34^ The fundamental frequency is crucial to provide LSI, which is needed for true quantitative imaging. In addition, the fundamental harmonic is needed to obtain the total point spread function (PSF), which can be employed in postprocessing to improve image quality. One method to recover the first harmonic is to estimate the lost baseline information of the MPI image from partial overlapping FOVs.^26^, ^27^ The combined passive and active drive field feed‐through compensation approach that is introduced here, does not need the acquisition of partial FOVs to recover the fundamental harmonic, but provides direct access to the particle signal at the fundamental excitation frequency. This is displayed by the system functions (Fig. 8) and the spectroscopic measurements (Fig. 6). The proposed compensation technique avoids elaborate recovery of the fundamental frequency and provides LSI properties. To the knowledge of the authors, the presented mf‐MPI is the first one‐dimensional MPI scanner that provides direct access to the first harmonic.

The reconstructed images (Fig. 10) prove the multifrequency imaging capability of the mf‐MPI. Phantom images were reconstructed successfully with all applied drive field frequencies (5, 7, 10, 13, and 15 kHz) using the acquired system matrices and the Kaczmarz approach. Two microliters of Perimag^®^ samples in a distance of 3 mm (center‐to‐center) could be resolved clearly. The full width at half maximum (FWHM) of the averaged reconstructions varying in the excitation frequencies with and without using the first harmonic was measured. The FWHM when using the first harmonic is 1.25 mm, compared to a FWHM of 1.48 mm when the fundamental frequency component is not employed. When omitting the fundamental harmonic of the system matrices in the reconstruction process, a noise increase by a factor of 1.72 can be observed in the reconstructed images and line profiles. Thus, the results indicate that the SNR can be increased by employing the fundamental harmonic. Small differences in location and width of the reconstructed images can be observed. However, the mentioned parameters do not seem to alter monotonically with the excitation frequency. One possible explanation is noise in the system, since no averaging was used for the system matrix acquisitions. The dip in the image of the left sample of the 15 kHz acquisition (Fig. 10) is as well most likely due to noise in the system matrix or image acquisitions. Note, that the selection field gradient of the scanner is only 0.7 T/m in x direction. By using a steeper gradient, the spatial resolution can be further increased.^5^

The proposed compensation method moreover enables the application of waveforms that are different from sinusoidal excitation as triangular, rectangular, ramps, or linear frequency sweeps. This will require the active compensation to be applicable for many frequency components, which will be implemented in the next prototype. Recent studies indicate the potential of these alternative drive field waveforms for MPI, as it can affect spatial resolution or reduce reconstruction computational load.^22^, ^35^, ^36^ The option to simultaneously apply multiple frequencies or use more than one frequency for image reconstruction further has great potential to increase image quality in terms of spatial resolution. A joint reconstruction (JR) approach as it is known from multicolor MPI^37^ could be used for image reconstruction if the acquisitions are performed separately. This will be investigated further during this project.

The first implementation of an experimental 1D mf‐MPI that allows a flexible choice of drive field frequencies up to 20 kHz for image acquisitions was presented here. Thus, a platform was developed that allows to further investigate the principle of multiparametric MPI by using SPIONs as local nanosensors. Recent studies have shown the potential of this technique, including MPI thermometry, estimating viscosity, Brownian/Néel contribution or assessing cell vitality.^9^, ^10^, ^11^, ^13^, ^14^

## 5 Conclusion

A novel 1D experimental mf‐MPI scanner was presented here. Broadband drive field feed‐through cancellation was provided by a combined passive and active compensation approach. Thus, drive field frequency flexibility from 0.5 to 20 kHz was enabled. In total, a combined drive field feed‐through suppression of up to −125 dB was achieved, which proved to be sufficient to allow image acquisitions. The technique further provides direct access to the fundamental frequency of the SPION signal. First images were acquired that demonstrate the feasibility of the presented mf‐MPI. Excitation frequency dependent modulation of the system functions were observed. An SNR enhancement by the factor of 1.7 was shown when the fundamental harmonic was employed during image reconstruction. In subsequent steps of this project, the presented scanner will be used to study the principle of multiparameter estimation by employing mf‐MPI.

## Conflict of Interest

The authors have no relevant conflict of interest to disclose.
